# Mediating Effects of Professional Quality of Life in the Association of Team Job Crafting With Career Success Among Nurses: A Structural Equation Model

**DOI:** 10.1155/jonm/6887124

**Published:** 2026-07-27

**Authors:** Yu Yan, Di-Fei Duan, Shu Gong, Deng-Yan Ma

**Affiliations:** ^1^ Department of Nephrology, Kidney Research Institute, West China Hospital/West China School of Nursing, Sichuan University, Chengdu 610000, Sichuan, China, scu.edu.cn; ^2^ Nursing Department, West China Hospital/West China School of Nursing, Sichuan University, Chengdu, Sichuan, China, scu.edu.cn

**Keywords:** career success, nurse, Professional Quality of Life, team job crafting

## Abstract

**Background:**

Team job crafting has emerged as an important organizational resource that may enhance nurses’ professional functioning. However, its potential mechanisms in shaping nurses’ career success remain insufficiently understood.

**Objective:**

This study examined the direct association between team job crafting and career success among nurses and explored the mediating roles of the three dimensions of Professional Quality of Life (ProQoL), including compassion satisfaction (CS), burnout (BO), and secondary traumatic stress (STS).

**Methods:**

This secondary data analysis used cross‐sectional survey data collected from a tertiary hospital in Sichuan Province, China, between January and March 2024. A total of 1870 nurses were included after data‐quality screening. Validated Chinese versions of the Team Job Crafting Scale for Nurses, ProQoL scale, and Career Success Scale were administered. Descriptive statistics, Pearson correlations, and structural equation modeling (SEM) with bias‐corrected bootstrapping were conducted to test the hypothesized mediation model.

**Results:**

Team job crafting was positively associated with nurses’ career success both directly (*β* = 0.113) and indirectly through two significant dimensions of ProQoL, CS and BO (total indirect effect *β* = 0.251, accounting for 69.0% of the total effect). Among these mediators, CS showed the strongest effect (*β* = 0.167; 45.9% of total effect), followed by BO (*β* = 0.095; 26.1% of total effect). In contrast, the mediating effect of STS was not significant (*β* = −0.011).

**Conclusion:**

Team job crafting is an important predictor of nurses’ career success, operating primarily through increased CS and reduced BO. STS, however, does not appear to mediate this relationship. These findings highlight the potential value of team‐based interventions that enhance CS and reduce BO to foster nurses’ career success. Supportive team environments that promote positive professional experiences and mitigate emotional strain may, therefore, contribute to nurses’ long‐term career development.

## 1. Introduction

Nurses play a fundamental role in ensuring care quality and patient safety within global healthcare systems [1]. Nursing work is inherently complex and high risk, requiring professionals to provide timely and accurate care in fast‐paced and demanding environments [2]. As a result, well‐designed job structures have become essential for promoting work engagement, enhancing team performance, and safeguarding the quality of nursing care [3]. Recent evidence has further shown that positive emotional and psychological resources are closely associated with nurses’ work engagement and work performance, underscoring the importance of resource‐supportive conditions in demanding care environments [4, 5]. Against this backdrop, the concept of “job crafting” has gained increasing attention [6]. In nursing, job crafting often refers to self‐initiated adjustments made by nurses to improve their skills, strengthen competence, and maintain professional motivation [7]. With the advancement of modern medicine, multidisciplinary teamwork has become the dominant model of nursing practice [8]. Nursing tasks rely heavily on resource sharing, information exchange, and collaborative decision‐making within teams. Accordingly, the adjustment of task boundaries and role responsibilities increasingly occurs at the team [9] rather than solely the individual level. In response to this shift, team job crafting [10] has emerged as a team‐level proactive process in which members collectively redesign the task, relational, and cognitive aspects of their work to optimize team functioning and better balance job demands and resources. Because this concept more closely reflects the collaborative and interdependent nature of nursing work [11], it has become an important focus in contemporary nursing management research.

Career success is an important indicator of employees’ long‐term professional development [12]. Compared with objective career success, such as promotion or salary growth, subjective career success has received increasing attention. It reflects individuals’ internal evaluations of their career progress and achievements, primarily comprising two core dimensions: career satisfaction and perceived career competitiveness [13]. For nurses, career satisfaction reflects their sense of value realization and contentment with their professional trajectory, while career competitiveness denotes their self‐assessed clinical competence and adaptability in a demanding healthcare environment [14]. A stronger sense of subjective career success is associated with greater work motivation and lower turnover intention, playing a vital role in maintaining a stable nursing workforce and ensuring high‐quality patient care [15–17].

Team job crafting may positively influence nurses’ subjective career success through its relevance to the two core dimensions of career success, namely, career competitiveness and career satisfaction. First, through collaborative task redesign, smoother information sharing, and mutual support, team job crafting can help nurses optimize task allocation, improve role coordination, and make better use of shared professional resources [18, 19]. These collaborative adjustments may strengthen nurses’ confidence in their professional competence and enhance their perceived career competitiveness [20, 21]. Second, by enabling nurses to actively reshape how work is organized and experienced within the team, team job crafting may also promote a greater sense of achievement, fit, and satisfaction with their career development [22]. Therefore, team job crafting may contribute to higher levels of subjective career success among nurses.

The Job Demands–Resources (JD‐R) model [23] posits that job resources stimulate motivational processes and support career development, whereas excessive job demands lead to strain and burnout (BO). Within this framework, proactive behaviors that increase resources or reduce unnecessary job demands may improve employees’ Professional Quality of Life (ProQoL) and, consequently, promote career success. Evidence suggests that a favorable work environment is an important correlate of nurses’ ProQoL [24, 25]. Team job crafting, as a typical resource‐enhancing strategy, enables nurses to obtain greater structural, social, and psychological resources [26, 27], while also helping optimize the team work environment. According to the JD–R model, these resources may enhance ProQoL by increasing compassion satisfaction (CS) and reducing BO and secondary traumatic stress (STS) [28]. In turn, higher levels of ProQoL may foster the emotional well‐being and work motivation necessary for long‐term professional development and career success [29].

Taken together, team job crafting may influence nurses’ career success, and ProQoL may serve as an important underlying pathway. However, few studies have examined whether the three dimensions of ProQoL—CS, BO, and STS—mediate the relationship between team job crafting and career success among nurses. Therefore, this study aimed to examine the association between team job crafting and career success and to further test the mediating roles of the three dimensions of ProQoL. The following hypotheses were proposed:

• H1: Team job crafting positively influences nurses’ career success;
• H2: ProQoL mediates the relationship between team job crafting and career success;
• H2a: CS mediates the relationship between team job crafting and career success;
• H2b: BO mediates the relationship between team job crafting and career success;
• H2c: STS mediates the relationship between team job crafting and career success.

**FIGURE 1:**
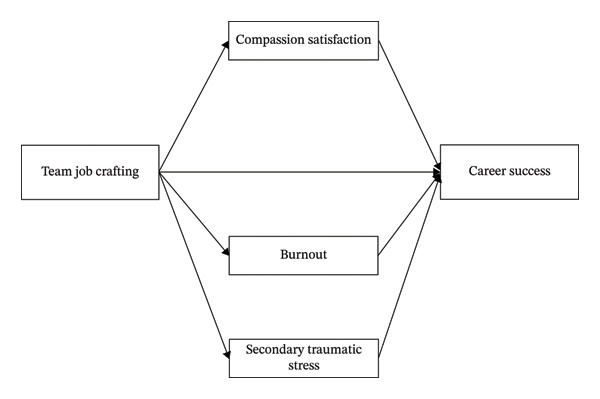
Research theoretical model.

Based on the above hypotheses, the present study formulated the research theoretical model shown in Figure 1.

## 2. Methods

### 2.1. Study Design, Setting, and Participants

This study employed a secondary data analysis approach using data collected from a previously conducted cross‐sectional study. The original study [29] was carried out between January and March 2024 in a tertiary general hospital located in Sichuan Province, China, which employs approximately 5000 nursing staff. Convenience sampling was used to recruit clinical nurses. Because convenience sampling was conducted in a single tertiary hospital, some degree of selection bias cannot be excluded. The inclusion criteria of the primary study were (1) being a registered nurse with a nationally recognized professional license, (2) having at least 1 year of employment, and (3) willingness to participate. Nurses who were on leave during the survey period due to pregnancy, illness, or personal reasons were excluded. With permission from the original research team, the present study conducted secondary analyses to examine the relationships among team job crafting, ProQoL, and career success. This dataset was selected because it included validated measures of all key constructs relevant to the present study, namely, team job crafting, ProQoL, and career success, in a large sample of clinical nurses.

The original dataset contained 2253 returned questionnaires. To ensure data quality, questionnaires were screened for evidence of insufficient‐effort responding based on response time and response‐pattern indicators. Specifically, questionnaires were excluded if the completion time was less than 6 min or if they showed fully invariant response patterns across all Likert‐type items, operationalized as zero variance in responses across those items. This response‐pattern screening was used as a conservative data‐quality check to identify only the most extreme cases of potentially invalid responding [30, 31]. After screening, 1870 questionnaires were retained for the final analyses.

### 2.2. Measurements

#### 2.2.1. Demographic Variables

Demographic variables included gender, age, marital status, education background, professional title, working area, rotational night shift, and willingness to continue nursing.

#### 2.2.2. Team Job Crafting Scale for Nurses (TJCS‐N)

Team job crafting was measured using the Chinese version of the TJCS‐N, originally developed by Iida et al. [9] and later translated and validated in Chinese by Fang et al. [32]. The scale contains 13 items rated on a five‐point Likert scale ranging from 1 (*not at all*) to 5 (*extremely*). The Chinese version comprises two dimensions: task crafting, which refers to collaborative adjustments in task allocation and work processes within the team, and cognitive and relational crafting, which refers to shared changes in how work is understood and how team members interact and support one another. Higher scores indicate higher levels of team job crafting among nurses. The instrument has demonstrated excellent internal consistency, with a Cronbach’s *α* coefficient of 0.95 reported for the Chinese version. In this study, the TJCS‐N demonstrated adequate reliability (Cronbach’s *α* = 0.976).

#### 2.2.3. ProQoL Scale

ProQoL was measured using the ProQoL scale developed by Stamm [33], which has been widely applied to assess the positive and negative professional experiences of nurses. The scale consists of 30 items and includes three subdimensions: CS, BO, and STS. CS reflects the sense of accomplishment, fulfillment, and meaning individuals gain from helping others. BO represents feelings of helplessness, frustration, and negative attitudes toward work that arise from prolonged workload and emotional exhaustion. STS describes the psychological stress reactions caused by repeated exposure to patients’ suffering or traumatic events. Items are rated on a six‐point Likert scale ranging from 0 (*never*) to 5 (*very often*). Each subscale contains 10 items, with higher scores indicating stronger experiences in that dimension. Specifically, higher scores on the CS subscale indicate a more positive ProQoL, whereas higher scores on the BO and STS subscales represent a poorer overall ProQoL. Previous studies reported a Cronbach’s *α* of 0.91 for the Chinese version of the ProQoL, with subscale coefficients ranging from 0.73 to 0.87 [34], demonstrating good internal consistency. In the present study, the Cronbach’s *α* values for the three subscales were 0.929, 0.842, and 0.834.

#### 2.2.4. Career Success Scale

The Career Success Scale was used to assess individuals’ positive psychological experiences and sense of achievement at work. The scale was originally developed by Eby et al. in 2003 [35]. Li et al. [14] later validated the scale among Chinese nurses and reported a Cronbach’s *α* of 0.87 for the total scale. The instrument consists of 11 items across two dimensions: career competitiveness (six items) and career satisfaction (five items). A five‐point Likert scale is used, ranging from 1 (*strongly disagree*) to 5 (*strongly agree*), with total scores ranging from 11 to 55. Higher scores indicate stronger perceived career success. In the present study, the Cronbach’s *α* for the overall scale was 0.915, demonstrating good internal consistency in our sample.

### 2.3. Data Analysis

The data were analyzed using SPSS 26.0 and AMOS 26.0. Descriptive statistics, including means, standard deviations, and frequencies, were used to summarize the sample characteristics and main study variables. Pearson correlation analyses were conducted to examine the bivariate relationships among team job crafting, ProQoL, and career success. Structural equation modeling (SEM) in AMOS was used to test the hypothesized mediation model. In the SEM, team job crafting was modeled as a latent variable indicated by task crafting and cognitive and relational crafting. Career success was modeled as a latent variable indicated by career competitiveness and career satisfaction. CS, BO and STS were entered as observed mediating variables based on their subscale scores. Mediation effects were assessed using the bias‐corrected bootstrap method with 5000 resamples, and significance was determined based on 95% confidence intervals (CI) that did not include zero. Standardized effect estimates (*β*) and their 95% bias‐corrected bootstrap confidence intervals were reported. Model fit was evaluated using multiple fit indices, including the chi‐square statistic to degrees of freedom ratio (*χ* ^2^/df), Goodness‐of‐Fit Index (GFI), Adjusted Goodness‐of‐Fit Index (AGFI), Normed Fit Index (NFI), Comparative Fit Index (CFI), Tucker–Lewis Index (TLI), Incremental Fit Index (IFI), and the Root Mean Square Error of Approximation (RMSEA). The following a priori criteria were used to evaluate model fit: *χ* ^2^/df ≤ 5.0, with values ≤ 3.0 indicating good fit; GFI, AGFI, NFI, CFI, TLI, and IFI ≥ 0.95; and RMSEA < 0.08 [36].

## 3. Results

### 3.1. Demographic Characteristics

Of the 1870 nurses included in the final analysis, 93.3% were female (*n* = 1745). The mean age was 33.17 ± 7.14 years. A total of 1601 participants held a bachelor’s degree. Most nurses (72.9%) held a junior professional title. Regular night‐shift rotation was common, reported by 1220 nurses (65.2%). Regarding career intentions, 39 nurses indicated they did not plan to remain in the nursing profession, and 385 reported being uncertain. Detailed demographic characteristics are presented in Table 1.

**TABLE 1:** Characteristics of participants (*N* = 1870).

Variables	*n* (%)
Gender	
Male	125 (6.7)
Female	1745 (93.3)
Age (years)	33.17 ± 7.14
Marital status	
Unmarried	611 (32.7)
Married	1216 (65.0)
Divorced/widowed	38 (2.0)
Education background	
Associate Degree	97 (5.2)
Bachelor’s Degree	1601 (85.6)
Master’s Degree	166 (8.9)
Doctoral Degree	6 (0.3)
Professional title	
Junior	1363 (72.9)
Intermediate	429 (22.9)
Senior	78 (4.2)
Working area	
Internal medicine	608 (32.5)
Surgery	432 (23.1)
Intensive care units	369 (19.7)
Other wards	461 (24.7)
Rotational night shift	
Yes	1220 (65.2)
No	650 (34.7)
Willingness to continue nursing	
Willing	1446 (77.3)
Unwilling	39 (2.0)
Not Sure	385 (20.5)

### 3.2. Descriptive Statistics and Bivariate Correlations

Table 2 presents the descriptive statistics and intercorrelations of the study variables. Overall, the mean scores were 22.94 ± 5.58 for BO, 36.65 ± 6.55 for CS, 24.21 ± 5.71 for STS, 55.22 ± 8.93 for team job crafting, and 38.78 ± 7.03 for career success. Correlation analysis showed clear patterns among the variables, and all correlations were statistically significant (*p* < 0.01). The strongest association was observed between BO and CS, which showed a strong negative correlation (*r* = −0.78). BO was also strongly positively correlated with STS (*r* = 0.60) and strongly negatively correlated with career success (*r* = −0.51), while showing a moderate negative correlation with team job crafting (*r* = −0.40). In addition, CS was strongly positively correlated with career success (*r* = 0.58) and moderately positively correlated with team job crafting (*r* = 0.39), but weakly negatively correlated with STS (*r* = −0.21). Team job crafting was moderately positively correlated with career success (*r* = 0.33) and weakly negatively correlated with STS (*r* = −0.20).

**TABLE 2:** Descriptive statistics and correlations among study variables.

Variables	(Min, max)	Mean (SD)	BO	CS	STS	TJC	CSS
BO	(10, 43)	22.94 ± 5.58	1				
CS	(10, 49)	36.65 ± 6.55	−0.78^∗∗^	1			
STS	(10, 45)	24.21 ± 5.71	0.60^∗∗^	−0.21^∗∗^	1		
TJC	(13, 65)	55.22 ± 8.93	−0.40^∗∗^	0.39^∗∗^	−0.20^∗∗^	1	
CSS	(14, 54)	38.78 ± 7.03	−0.51^∗∗^	0.58^∗∗^	−0.16^∗∗^	0.33^∗∗^	1

*Note:* BO: burnout.

Abbreviations: CS, compassion satisfaction; CSS, Career Success Scale; STS, secondary traumatic stress; TJC, team job crafting.

^∗∗^ *p* < 0.01.

Given the strong negative correlation between BO and CS, supplementary collinearity diagnostics were conducted prior to the mediation analysis. The maximum variance inflation factor (VIF) was 5.133 (for BO), and the lowest tolerance was 0.195. Although the VIF for BO slightly exceeded the stricter threshold of 5, all VIF values were below the commonly accepted cutoff of 10 [37], suggesting that multicollinearity was within an acceptable range and did not appear to pose a severe threat to model estimation.

### 3.3. SEM

**FIGURE 2:**
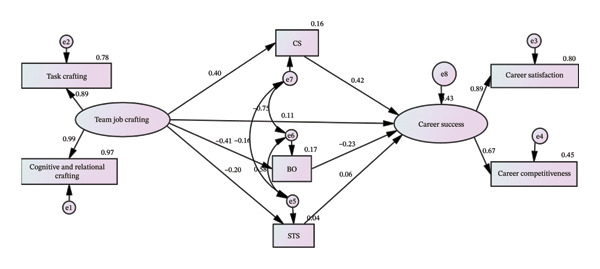
The validated model.

SEM was conducted to examine the hypothesized relationships among team job crafting, ProQoL components (CS, BO, and STS), and career success. In the parallel mediation model, the residual terms of CS, BO, and STS were allowed to covary to account for their shared variance. The standardized path coefficients of the final model are presented in Figure 2.

The overall model demonstrated a satisfactory fit to the data. As shown in Table 3, the *χ* ^2^/df ratio was 5.599. Although the *χ* ^2^/df ratio slightly exceeded the conventional threshold, the chi‐square statistic is known to be sensitive to the sample size. In large samples, even relatively small model–data discrepancies may lead to larger chi‐square values [38]. Therefore, model fit was further evaluated using additional fit indices. The incremental and absolute fit indices were all favorable, with GFI = 0.991, AGFI = 0.965, TLI = 0.953, CFI = 0.984, NFI = 0.981, and IFI = 0.984. The RMSEA was 0.050, which was below the recommended cutoff of 0.08. Collectively, these indices indicate that the proposed SEM model adequately fit the observed data.

**TABLE 3:** Applicability index of the structural equation model.

Fit metrics	Acceptable ranges	The present model fits	Results
*χ* ^2^/df	≤ 5 acceptable; ≤ 3 good	5.599	Slightly high but acceptable
GFI	≥ 0.95	0.991	Excellent
AGFI	≥ 0.95	0.965	Excellent
TLI	≥ 0.95	0.953	Excellent
CFI	≥ 0.95	0.984	Excellent
NFI	≥ 0.95	0.981	Excellent
IFI	≥ 0.95	0.984	Excellent
RMSEA	< 0.08	0.050	Excellent

### 3.4. Mediating Role Analysis

As shown in Table 4, the direct effect of team job crafting on career success was significant (*β* = 0.113 and 95% CI: 0.055–0.167), accounting for 31.0% of the total effect. The total indirect effect was also significant (*β* = 0.251 and 95% CI: 0.214–0.287), explaining 69.0% of the overall association between team job crafting and career success.

**TABLE 4:** Mediation effects of ProQoL between team job crafting and career success.

Path	Effect (*β*)	Boot SE	*p*	Bootstrap 95% CI		Effect ratio (%)
				Low	High	
Total effect	0.364	0.033	< 0.001	0.291	0.421	100.0
Direct effect (c′)	0.113	0.029	< 0.001	0.055	0.167	31.0
Total indirect effect	0.251	0.018	< 0.001	0.214	0.287	69.0
Ind1: TJC ⟶ CS ⟶ CSS	0.167	0.021	< 0.001	0.128	0.213	45.9
Ind2: TJC ⟶ BO ⟶ CSS	0.095	0.022	< 0.001	0.053	0.139	26.1
Ind3: TJC⟶ STS ⟶ CSS	−0.011	0.007	0.074	−0.026	0.001	−3.0

*Note:* Effect ratios were calculated as the ratio of each indirect effect to the total effect, multiplied by 100. BO: burnout.

Abbreviations: CS, compassion satisfaction; CSS, career success scale; STS, secondary traumatic stress; TJC, team job crafting.

Among the three mediating pathways, the indirect effect through CS (team job crafting ⟶ CS ⟶ career success) was the strongest (*β* = 0.167 and 95% CI: 0.128–0.213), contributing 45.9% of the total effect. The pathway through BO (team job crafting ⟶ BO ⟶ career success) was also statistically significant (*β* = 0.095 and 95% CI: 0.053–0.139), accounting for 26.1% of the total effect. In contrast, the indirect effect through STS (team job crafting ⟶ STS ⟶ career success) was not significant (*β* = −0.011 and 95% CI: −0.026–0.001), as the 95% CI included zero.

## 4. Discussion

This study provides new insights into how team job crafting dynamics influence nurses’ professional development. By examining team job crafting together with key components of ProQoL, our findings reveal a clear and consistent pattern: nurses who engage in higher levels of team job crafting report greater career success. This association is largely explained by two psychological pathways—enhanced CS and reduced BO—whereas STS did not demonstrate a significant mediating effect.

A moderate level of career success was observed among nurses in this study (mean = 38.78, SD = 7.03), consistent with previous findings in Chinese nursing populations [39, 40]. Within the Chinese healthcare context, such a moderate level may reflect the combined influence of cumulative work fatigue [41], relatively limited social recognition of the nursing profession [42], tensions in nurse–patient relationships [43], and the demanding service environment in hospitals [44]. These contextual pressures may be especially salient in tertiary hospitals [45], where nurses commonly work under high‐intensity conditions characterized by heavier workloads, larger patient volumes, greater disease severity, and more complex interpersonal environments [46]. Given that the present study was conducted in a tertiary hospital, these contextual and system‐related features of Chinese nursing practice may partly explain the moderate level of career success found in our sample. Accordingly, it is crucial to identify organizational resources that can mitigate these pressures and promote nurses’ professional development in high‐pressure tertiary care settings.

Our findings supported Hypothesis 1, showing that team job crafting is positively associated with nurses’ career success. Previous studies have linked team job crafting with enhanced work engagement and career well‐being [18, 47]. Our study extends this by highlighting its association with subjective career success—a broader reflection of professional growth, recognition, and accomplishment. In team‐oriented nursing settings, team job crafting allows nurses to adjust roles, coordinate tasks, and improve their work environment through collaboration [10, 48]. This approach is especially meaningful in China, where high patient volumes and complex workflows often require efficient teamwork. In such contexts, higher levels of team job crafting may be associated with greater role clarity, stronger perceived support, and more positive perceptions of one’s professional development [49–51]. Accordingly, nurse managers may consider fostering a supportive team environment, encouraging nurses’ participation in team job crafting, and recognizing collective contributions. Such efforts may be helpful approaches for higher career satisfaction and more positive professional development experiences.

Our findings partially supported Hypothesis 2. In addition to a direct effect, team job crafting showed a significant indirect effect on career success through ProQoL, accounting for 69.0% of the total effect. This highlights ProQoL as a key mechanism linking team job crafting to nurses’ long‐term professional development.

Among the three dimensions of ProQoL, CS was the strongest mediator, explaining 45.9% of the total effect. This supports Hypothesis 2a. Previous studies have shown a significant positive association between team job crafting and CS among nurses [28]. By adjusting task roles, cognitive appraisals, and relational interactions, team job crafting can facilitate more effective allocation of team resources, alleviate role ambiguity–related cognitive strain, and reduce nurses’ physical and emotional fatigue. These improvements may enable nurses to devote greater energy to compassionate patient care [52]. And nurses’ CS has been shown to positively influence a range of favorable professional outcomes, such as job satisfaction and work performance, which in turn contribute to higher levels of subjective career success [53]. Taken together, these findings suggest that CS serves as an important positive mechanism through which team job crafting enhances nurses’ career success. Notably, this crucial mediating role of CS aligns with broader evidence from other countries and regions, where CS has been consistently identified as a key driver of nurse retention [54, 55], job satisfaction [56]. Therefore, improving CS through team job crafting may be an effective way to promote nurses’ career success, especially in high‐pressure clinical environments.

BO also served as a significant mediator, accounting for 26.1% of the total effect, which supports Hypothesis 2b. Consistent with previous findings [28], higher levels of team job crafting were significantly associated with lower levels of BO among nurses. According to the JD–R model [57], team job crafting can increase job resources, such as task autonomy, coworker support, and role clarity. It can also reduce unnecessary hindrance demands. These changes may buffer the adverse effects of high job demands on nurses and thereby lower the risk of burnout. In addition, in a well‐organized and collaborative team environment, nurses may regulate their emotional investment and work pace more proactively and find opportunities for recovery even under high work intensity [58]. Recent evidence from high‐stress nursing settings [59] also suggests that more effective coping may help nurses better manage workplace stress, which provides additional context for understanding why team job crafting may reduce BO by strengthening team support, coordination, and role clarity. This adaptive process may help them achieve a better balance between workload and recovery needs, thereby reducing the risk of emotional exhaustion [60]. Conversely, failing to manage such emotional exhaustion can severely hinder professional growth. According to the three‐dimensional model of occupational BO [61], BO comprises emotional exhaustion, depersonalization, and reduced personal accomplishment. BO depletes nurses’ emotional and cognitive resources, diminishes work engagement, impairs interactions with patients and colleagues, and lowers professional achievement and self‐efficacy [62, 63]. These impairments can undermine job performance and motivation for career development, ultimately exerting a negative influence on nurses’ career success.

In contrast, STS did not significantly mediate the relationship between team job crafting and career success, thus Hypothesis 2c was not supported. One possible explanation is that STS is typically associated with acute traumatic exposure and emotionally charged clinical situations, which are more prevalent in departments such as emergency, critical care, or oncology [64]. As our study involved a hospital‐wide sample covering diverse units rather than focusing on high‐risk specialties, the overall level and variability of STS may have been insufficient to reveal a significant effect. Given that STS is more situational and event‐driven, future research should examine high‐risk units separately, particularly emergency departments, intensive care units, and oncology settings, to determine whether STS plays a more meaningful mediating role in these clinical contexts.

### 4.1. Limitations

This study has several limitations that should be acknowledged. First, the cross‐sectional design precludes establishing causal relationships among team job crafting, ProQoL, and career success. Longitudinal or intervention‐based studies are required to establish temporal precedence and validate the proposed mechanisms. Second, all variables were measured using self‐reported questionnaires, which may introduce common method bias and social desirability effects. Future studies could incorporate multisource assessments, such as peer or supervisor evaluations, or use more objective measures to reduce these potential biases. In addition, although the TJCS‐N is a well‐established instrument and its Chinese version has demonstrated good reliability and validity, the very high internal consistency observed in the present study (Cronbach’s *α* = 0.976) may suggest substantial overlap among some items in this sample. Future research may further examine the scale structure and evaluate whether a shortened version could retain adequate psychometric performance. Third, some potentially important variables were not included in the present study, such as the leadership style, unit staffing levels, and other organizational factors, which may confound the observed associations. Future research should incorporate these factors to provide a more comprehensive understanding of the relationships among the study variables. Finally, the data were collected from nurses in a single tertiary hospital, which may limit the generalizability of the results. Future research should include multicenter samples across diverse hospital settings to enhance external validity and improve the robustness of the conclusions.

## 5. Conclusion

This study showed that team job crafting was directly and indirectly associated with nurses’ career success, mainly through higher CS and lower BO, whereas STS was not a significant mediating pathway. From a practical perspective, possible supportive actions may include regular team‐based task adjustment meetings, peer recognition and feedback mechanisms, and periodic BO assessments to inform staffing, workload distribution, and resource allocation. Together, these efforts may help create a more supportive team environment, strengthen CS, alleviate BO, and promote more positive perceptions of career success among nurses.

## Author Contributions

Yu Yan: conceptualization, software, methodology, formal analysis, writing–original draft, and writing–review and editing; Di‐Fei Duan: conceptualization, methodology, formal analysis, writing–original draft, and writing–review and editing; Shu Gong: conceptualization, methodology, supervision, and writing–review and editing; Deng‐Yan Ma: writing–review and editing, methodology, conceptualization, and supervision.

## Funding

No external funding was received for this study.

## Ethics Statement

Ethical approval for this study was obtained from the Ethics Committee of West China Hospital, Sichuan University (no. 2024105). Informed consent was obtained from all participants involved in the study.

## Conflicts of Interest

The authors declare no conflicts of interest.

## Data Availability

The data that support the findings of this study are available from the corresponding author upon reasonable request.

## References

[1] Pintye J. , Update on the State of the World’s Nursing, MCN: The American Journal of Maternal/Child Nursing. (2025) 50, no. 6, 10.1097/nmc.0000000000001137. 41092472

[2] Mcvicar A. , Workplace Stress in Nursing: a Literature Review, Journal of Advanced Nursing. (2003) 44, no. 6, 633–642, 10.1046/j.0309-2402.2003.02853.x. 14651686

[3] Topa G. and Aranda-Carmena M. , Job Crafting in Nursing: Mediation Between Work Engagement and Job Performance in a Multisample Study, International Journal of Environmental Research and Public Health. (2022) 19, no. 19, 10.3390/ijerph191912711. PMC9566469 36232011

[4] Aqtam I. , Ayed A. , Batran A. , Ejheisheh M. A. , Melhem R. H. , and Shouli M. , Work Engagement and Its Association with Emotional Intelligence and Demographic Characteristics Among Nurses in Palestinian Neonatal Intensive Care Units, PLoS One. (2025) 20, no. 9, 10.1371/journal.pone.0332908. PMC12448998 40971854

[5] Ayed A. , The Relationship Between the Emotional Intelligence and Clinical Decision-Making Among Nurses in Neonatal Intensive Care Units, SAGE Open Nurs. (2025) 11, 10.1177/23779608251321352. PMC11846121 39990062

[6] Oldham G. R. and Hackman J. R. , Not what it Was and Not what it Will be: the Future of Job Design Research, Journal of Organizational Behavior. (2010) 31, no. 2‐3, 463–479, 10.1002/job.678.

[7] Yuan X. , Yin X. , Zhang X. et al., Job Crafting in Nursing: a Conceptual Analysis for Theoretical Advancements in Nursing Practice, Journal of Nursing Management. (2025) . 10.1155/jonm/6599866 PMC12375838 40860614

[8] Cheng H. , Ding Y. , and Wang B. , A Validation Study of the Job Crafting Scale Among Nurses in Public Hospitals in China, Journal of Nursing Management. (2020) 28, no. 5, 1021–1029, 10.1111/jonm.12998. 32145121

[9] Iida M. , Watanabe K. , Imamura K. et al., Development and Validation of the Japanese Version of the Team Job Crafting Scale for Nurses, Research in Nursing & Health. (2021) 44, no. 2, 329–343, 10.1002/nur.22110. 33512763

[10] Goh P. Q. L. , Ser T. F. , Cooper S. , Cheng L. J. , and Liaw S. Y. , Nursing Teamwork in General Ward Settings: a mixed-methods Exploratory Study Among Enrolled and Registered Nurses, Journal of Clinical Nursing. (2020) 29, no. 19-20, 3802–3811, 10.1111/jocn.15410. 32643794

[11] Iida M. , Sakuraya A. , Watanabe K. et al., The Association Between Team Job Crafting and Work Engagement Among Nurses: a Prospective Cohort Study, BMC Psychology. (2024) 12, no. 1, 10.1186/s40359-024-01538-7. PMC10854162 38336755

[12] Brownrout J. , Norato G. , Bensken W. et al., Influence of Research Continuity on Physician-Scientists’ Career Success, Neurology. (2021) 97, no. 20, e2039–e2045, 10.1212/wnl.0000000000012867. 34670817 PMC8672432

[13] Chang P. C. , Guo Y. , Cai Q. , and Guo H. , Proactive Career Orientation and Subjective Career Success: a Perspective of Career Construction Theory, Behavioral Sciences. (2023) 13, no. 6, 10.3390/bs13060503. PMC10295766 37366755

[14] Li Z. K. , You L. M. , Lin H. S. , and Chan S. W. , The Career Success Scale in Nursing: Psychometric Evidence to Support the Chinese Version, Journal of Advanced Nursing. (2014) 70, no. 5, 1194–1203, 10.1111/jan.12285. 24304445

[15] Zhang Y. , An Y. , Wang L. , Zhao Q. , Li H. , and Fan X. , Psychosocial Factors Associated with Career Success Among Nurses: a Latent Profile Analysis, Journal of Advanced Nursing. (2023) 79, no. 2, 652–663, 10.1111/jan.15524. 36484162

[16] Wu C. , Fu M. M. , Cheng S. Z. et al., Career Identity and Career Success Among Chinese Male Nurses: the Mediating Role of Work Engagement, Journal of Nursing Management. (2022) 30, no. 7, 3350–3359, 10.1111/jonm.13782. 36056581 PMC10087454

[17] Ejheisheh M. A. , Ayed A. , Salameh B. et al., Understanding the Relationship Between Professional Values and Caring Behavior Among Nurses in Intensive Care Units: a cross-sectional Study from Palestine, BMC Nursing. (2025) 24, no. 1, 10.1186/s12912-025-02903-6. PMC11917032 40102898

[18] Wu L. , Sui W. , Xia Y. et al., The Mediating Effect of Team Job Crafting on the Association Between Transformational Leadership and Occupational Well-Being in Newly Graduated Nurses, Journal of Advanced Nursing. (2025) . 10.1111/jan.70313 41201001

[19] Tims M. , Bakker A. B. , Derks D. , and van Rhenen W. , Job Crafting at the Team and Individual Level:Implications for Work Engagement and Performance, Group & Organization Management. (2013) 38, no. 4, 427–454, 10.1177/1059601113492421.

[20] Hung T.-K. , Wang C.-H. , Tian M. , and Yang Y. J. , A Cross-Level Investigation of Team-Member Exchange on Team and Individual Job Crafting with the Moderating Effect of Regulatory Focus, International Journal of Environmental Research and Public Health. (2020) 17, no. 6, 10.3390/ijerph17062044. PMC7143217 32204448

[21] De Jong J. P. , De Clippeleer I. , and De Vos A. , Enhancing Team Crafting Through Proactive Motivation: an Intervention Study, Journal of Applied Psychology. (2025) 110, no. 2, 282–296, 10.1037/apl0001220. 39325376

[22] Hyun M. S. , The Impact of Grit and Job Crafting on Organizational Commitment and Job Satisfaction Among Hospital Nurses in Korea, Medicine (Baltimore). (2025) 104, no. 45, 10.1097/md.0000000000045890. PMC12599759 41248695

[23] Bakker A. B. and Demerouti E. , Job demands-resources Theory: Frequently Asked Questions, Journal of Occupational Health Psychology. (2024) 29, no. 3, 188–200, 10.1037/ocp0000376. 38913705

[24] Batran A. , Aqtam I. , Ayed A. , and Ejheisheh M. A. , The Relationship Between Professional Quality of Life and Work Environment Among Nurses in Neonate Care Units, PLoS One. (2025) 20, no. 4, 10.1371/journal.pone.0322023. PMC12027106 40279366

[25] Ayed A. , Abu Ejheisheh M. , Aqtam I. , Batran A. , and Farajallah M. , The Relationship Between Professional Quality of Life and Work Environment Among Nurses in Intensive Care Units, Inquiry. (2024) 61, 10.1177/00469580241297974. PMC11550503 39520216

[26] Xu Z. , Zhao B. , Zhang Z. et al., Prevalence and Associated Factors of Secondary Traumatic Stress in Emergency Nurses: a Systematic Review and meta-analysis, European Journal of Psychotraumatology. (2024) 15, no. 1, 10.1080/20008066.2024.2321761. PMC10911249 38426665

[27] Tims M. , Bakker A. B. , Derks D. , and van Rhenen W. , Job Crafting at the Team and Individual Level: Implications for Work Engagement and Performance, Group & Organization Management. (2013) 38, no. 4, 427–454, 10.1177/1059601113492421.

[28] Zhou X. H. , Duan D. F. , Chen L. , Zhang Y. J. , Gong S. , and Chen Q. , The Effect of Team Job Crafting on Professional Quality of Life Among Nurses: a Latent Profile Analysis, Journal of Nursing Management. (2025) 2025, no. 1, 10.1155/jonm/2320459. PMC12283198 40697332

[29] Zhou X. H. , Duan D. F. , Chen L. , Zhang Y. , Gong S. , and Chen Q. , Relationship Between Professional Quality of Life and Career Success in Nurses: a Latent Profile Analysis, International Nursing Review. (2025) 72, no. 2, 10.1111/inr.70044. 40528567

[30] Ward M. K. and Meade A. W. , Dealing with Careless Responding in Survey Data: Prevention, Identification, and Recommended Best Practices, Annual Review of Psychology. (2023) 74, no. 1, 577–596, 10.1146/annurev-psych-040422-045007. 35973734

[31] Curran P. G. , Methods for the Detection of Carelessly Invalid Responses in Survey Data, Journal of Experimental Social Psychology. (2016) 66, 4–19, 10.1016/j.jesp.2015.07.006.

[32] Fang R. , Wu G. , Yan W. et al., Chinese Adaptation and Reliability and Validity Test of the Nurse Teamwork Reshaping Scale, Chinese Nursing Management. (2024) 24, no. 1, 126–129.

[33] Stamm B. H. , The Proqol (Professional Quality of Life Scale: Compassion Satisfaction and Compassion Fatigue), 2010, ProQOL org.

[34] Zheng X. , Yang M. , Gao W. et al., Reliability and Validity Test of Chinese Version of Nurses’ Professional Quality of Life Scale, Journal of Nursing. (2013) 28, no. 05, 13–15.

[35] Zhang L. , Liang X. , Cheng N. et al., Psychological Resilience Mediates Sense of Professional Mission and Career Success in Chinese Intensive Care Unit Nurses: a cross-sectional Study, BMC Nursing. (2024) 23, no. 1, 10.1186/s12912-024-02271-7. PMC11367826 39218871

[36] Hu L. T. and Bentler P. M. , Cutoff Criteria for Fit Indexes in Covariance Structure Analysis: Conventional Criteria Versus New Alternatives, Structural Equation Modeling: A Multidisciplinary Journal. (1999) 6, no. 1, 1–55, 10.1080/10705519909540118.

[37] Kim J. H. , Multicollinearity and Misleading Statistical Results, Korean Journal of Anesthesiology. (2019) 72, no. 6, 558–569, 10.4097/kja.19087. 31304696 PMC6900425

[38] West S. G. , Taylor A. B. , and Wu W. , Model Fit and Model Selection in Structural Equation Modeling, Handbook of Structural Equation Modeling. (2012) 1, no. 1, 209–231.

[39] Xu H. , Cao X. , Jin Q. X. , Wang R. , Zhang Y. , and Chen Z. , The Impact of the Second Victim’s Experience and Support on the Career Success of Psychiatric Nurses: the Mediating Effect of Psychological Resilience, Journal of Nursing Management. (2022) 30, no. 6, 1559–1569, 10.1111/jonm.13467. 34435707

[40] Fu H. , Geng Y. , Zheng X. , and Wang A. , Mediating Effect of Work Engagement and Career Success on work-family Support and Turnover Intention of Hemodialysis Nurses in China: a cross-section Study, BMC Nursing. (2025) 24, no. 1, 10.1186/s12912-025-03223-5. PMC12105381 40420063

[41] Huang H. , Liu L. , Yang S. , Cui X. , Zhang J. , and Wu H. , Effects of Job Conditions, Occupational Stress, and Emotional Intelligence on Chronic Fatigue Among Chinese Nurses: a cross-sectional Study, Psychology Research and Behavior Management. (2019) 12, 351–360, 10.2147/prbm.s207283. 31191056 PMC6526330

[42] Zhou H. , Jiang F. , Rakofsky J. et al., Job Satisfaction and Associated Factors Among Psychiatric Nurses in Tertiary Psychiatric Hospitals: Results from a Nationwide cross-sectional Study, Journal of Advanced Nursing. (2019) 75, no. 12, 3619–3630, 10.1111/jan.14202. 31566793

[43] Wang Z. , Zhou Z. , Liu G. , Fan H. , Zhuang Y. , and Zhai X. , Mitigating Nurse Turnover in Urban China: Income Inequality and Nurse-Patient Relationships as Moderators of Occupational Stress, Journal of Advanced Nursing. (2026) 82, no. 4, 3272–3286, 10.1111/jan.70118. 40922523

[44] Zhang C. , Xu W. , Zhu L. , and Liao C. , Relationships Between Psychological Capital, Quality of Work Life and Career Success Among Nurse Managers in Tertiary General Hospitals: A Cross-Sectional Study, BMC Nursing. (2025) 24, no. 1, 10.1186/s12912-025-03854-8. PMC12465340 41013608

[45] Wu C. , Zhang L. Y. , Zhang X. Y. et al., Factors Influencing Career Success of Clinical Nurses in Northwestern China Based on Kaleidoscope Career Model: Structural Equation Model, Journal of Nursing Management. (2022) 30, no. 2, 428–438, 10.1111/jonm.13499. 34704641 PMC9298989

[46] 36th International Symposium on Intensive Care and Emergency Medicine: Brussels, Belgium, Critical Care. (2016) 20, no. Suppl 2. 10.1186/s13054-016-1208-6 PMC5493079 27885969

[47] Tuncer Unver G. , Celebi Cakiroglu O. , and Boduc N. , Team Job Crafting as a Mediator Between Sparking Leadership and Work Engagement Among Nurses: a Structural Equation Modeling Approach, Journal of Health Organism Management. (2025) 1–16, 10.1108/jhom-05-2025-0255. 41157937

[48] Zhang H. L. , Liu J. H. , Ma W. J. , Xu X. , Guo X. , and Lang H. , The Relationship Between Job Crafting and Work Engagement Among Nurses in China: a Latent Profile Analysis, Nursing Open. (2024) 11, no. 10, 10.1002/nop2.70007. PMC11495650 39437201

[49] Rafiq M. , Farrukh M. , Attiq S. , Shahzad F. , and Khan I. , Linking Job Crafting, Innovation Performance, and Career Satisfaction: the Mediating Role of Work Engagement, Work. (2023) 75, no. 3, 877–886, 10.3233/wor-211363. 36683474

[50] Trillo A. , Ortega-Maldonado A. , Lopez-Pena B. , and Bretones F. D. , Psychosocial Predictors of Job Satisfaction in Nursing: Insights from a Spanish Hospital Setting, Behavioral Sciences. (2025) 15, no. 3, 10.3390/bs15030274. PMC11939514 40150169

[51] Ding J. and Wu Y. , The Mediating Effect of Job Satisfaction and Emotional Exhaustion on the Relationship Between Psychological Empowerment and Turnover Intention Among Chinese Nurses During the COVID-19 Pandemic: a cross-sectional Study, BMC Nursing. (2023) 22, no. 1, 10.1186/s12912-023-01357-y. PMC10294374 37370072

[52] Ruiz-FernáNDEZ M. D. , Ramos-Pichardo J. D. , IbañEZ-Masero O. , Sánchez-Ruiz M. J. , Fernández-Leyva A. , and Ortega-Galán ÁM. , Perceived Health, Perceived Social Support and Professional Quality of Life in Hospital Emergency Nurses, International Emergency Nursing. (2021) 59, 10.1016/j.ienj.2021.101079. 34758447

[53] Bellicoso D. , Trudeau M. , Fitch M. I. , and Ralph M. R. , Chronobiological Factors for Compassion Satisfaction and Fatigue Among Ambulatory Oncology Caregivers, Chronobiology International. (2017) 34, no. 6, 808–818, 10.1080/07420528.2017.1314301. 28430534

[54] Jin-Hwa P. and Eun-Kyung L. , The Relationship Between leader-member Exchange and Intention to Stay in Korean Nurses: Focusing on the Mediating Role of Compassion Satisfaction, Nursing Practice Today. (2020) 8, no. 2.

[55] Wei H. , Cao Y. , Carroll Q. et al., Nursing Work Engagement, Professional Quality of Life, and Intent to Leave: a Structural Equation Modeling Pathway Analysis, Journal of Nursing Research. (2024) 32, no. 5, 10.1097/jnr.0000000000000632. 39324932

[56] Kim Y. H. , Kim S. R. , Kim Y. O. , and Kim J. Y. , Influence of Type D Personality on Job Stress and Job Satisfaction in Clinical Nurses: the Mediating Effects of Compassion Fatigue, Burnout, and Compassion Satisfaction, Journal of Advanced Nursing. (2017) 73, no. 4, 905–916, 10.1111/jan.13177. 27706839

[57] Bakker A. B. and Demerouti E. , Job demands-resources Theory: Taking Stock and Looking Forward, Journal of Occupational Health Psychology. (2017) 22, no. 3, 273–285, 10.1037/ocp0000056. 27732008

[58] Labrague L. J. and De Los Santos J. A. A. , COVID-19 Anxiety Among front-line Nurses: Predictive Role of Organisational Support, Personal Resilience and Social Support, Journal of Nursing Management. (2020) 28, no. 7, 1653–1661, 10.1111/jonm.13121. 32770780 PMC7436313

[59] Ayed A. , The Relationship Between Emotional Intelligence and Coping Behaviors Among Nurses in the Neonatal Intensive Care Unit, SAGE Open Nursing. (2025) 11, 10.1177/23779608251330097. PMC11920997 40109576

[60] Lee H. F. , Hsu H. C. , Efendi F. , Ramoo V. , and Susanti I. A. , Burnout, Resilience, and Empowerment Among COVID-19 Survivor Nurses in Indonesia, PLoS One. (2023) 18, no. 10, 10.1371/journal.pone.0291073. PMC10564161 37816005

[61] Bu T. , Peng C. , Liu J. et al., Nurse Burnout: Deep Connections and Solutions Revealed by Network Analysis, BMC Nursing. (2024) 23, no. 1, 10.1186/s12912-024-02190-7. PMC11297739 39095727

[62] Dulko D. and Kohal B. J. , How Do We Reduce Burnout in Nursing?, Nursing Clinical North America. (2022) 57, no. 1, 101–114, 10.1016/j.cnur.2021.11.007. 35236601

[63] Li L. Z. , Yang P. , Singer S. J. , Pfeffer J. , Mathur M. B. , and Shanafelt T. , Nurse Burnout and Patient Safety, Satisfaction, and Quality of Care: a Systematic Review and Meta-Analysis, JAMA Network Open. (2024) 7, no. 11, 10.1001/jamanetworkopen.2024.43059. PMC11539016 39499515

[64] Morrison L. E. and Joy J. P. , Secondary Traumatic Stress in the Emergency Department, Journal of Advanced Nursing. (2016) 72, no. 11, 2894–2906, 10.1111/jan.13030. 27221701

